# Controlling viral outbreaks: Quantitative strategies

**DOI:** 10.1371/journal.pone.0171199

**Published:** 2017-02-10

**Authors:** Anna Mummert, Howard Weiss

**Affiliations:** 1 Mathematics Department, Marshall University, Huntington, WV, United States of America; 2 School of Mathematics, Georgia Institute of Technology, Atlanta, GA, United States of America; The University of Melbourne, AUSTRALIA

## Abstract

Preparing for and responding to outbreaks of serious livestock infectious diseases are critical measures to safeguard animal health, public health, and food supply. Almost all of the current control strategies are empirical, and mass culling or “stamping out” is frequently the principal strategy for controlling epidemics. However, there are ethical, ecological, and economic reasons to consider less drastic control strategies. Here we use modeling to quantitatively study the efficacy of different control measures for viral outbreaks, where the infectiousness, transmissibility and death rate of animals commonly depends on their viral load. We develop a broad theoretical framework for exploring and understanding this heterogeneity. The model includes both direct transmission from infectious animals and indirect transmission from an environmental reservoir. We then incorporate a large variety of control measures, including vaccination, antivirals, isolation, environmental disinfection, and several forms of culling, which may result in fewer culled animals. We provide explicit formulae for the basic reproduction number, *R*_0_, for each intervention and for combinations. We evaluate the control methods for a realistic simulated outbreak of low pathogenic avian influenza on a mid-sized turkey farm. In this simulated outbreak, culling results in more total dead birds and dramatically more when culling all of the infected birds.

## Introduction

Zoonotic infections are estimated to cause more than two million human deaths every year [1]. Of particular concern are infections affecting livestock, because of close human interaction, zoonotic potential, and economic impact. Preparing for and responding to serious livestock epidemics are critical to safeguarding public health, animal health, and food supply.

Viral diseases in livestock with high economic and social impact, such as highly pathogenic avian influenza (HPAI), classical swine fever, African swine fever, and foot-and-mouth disease (FMD), require efficient control measures to prevent continued spread. No curative treatment exists for these diseases; eradications of local outbreaks are frequently based on mass culling combined with movement restrictions. These control measures are often implemented empirically, though it has long been argued that modeling should be more extensively used as a tool in veterinary epidemiology [2].

Mass culling (or “stamping out”) is the euthanasia of infected animals and all susceptible animals that have potentially been exposed to the virus on an infected premise, and in rare cases on other premises considered at risk. However, there are ethical, ecological, and economic reasons to consider less drastic control strategies. Animal welfare in disease outbreaks is receiving considerable attention as mass cullings have raised concerns over humane options for effected animals [3, 4]. Additionally, although mass culling may provide a useful short-term benefit, there could be serious evolutionary repercussions on reservoir hosts and the viral population. For instance, mass culling may impede the evolution of host resistance and select for heightened virulence and transmissibility [5].

During the 2001 outbreak of FMD in Britain, the government slaughtered nearly 6.5 million sheep, cattle, and pigs [6]. Not only were all the animals on infected farms culled, but also all animals on adjacent farms, regardless of whether infection had been reported there. Although there were a few models to support these actions, some veterinarians thought such mass depopulation was unnecessary (see [7] for an overview). They believed a more targeted approach would have been sufficient to end the outbreak, but they lacked models to assess alternative strategies. The model presented in [8] attempts to manage real-time outbreaks of FMD. One subsequent modeling study indicated that the outbreak could have been controlled by culling only animals known to be infected along with early detection of the outbreak [9].

In 2005, the United Nations Food and Agriculture Organization and World Organization for Animal Health announced that vaccination and segregation should replace mass culling during outbreaks of highly pathogenic avian influenza in developing countries [10]. This shift was driven by the realization that H5N1 is widespread in wild and domestic bird populations, and since bio-containment from wild birds would be prohibitively expensive, regardless of how often affected poultry flocks are culled, the virus is likely to reappear. Also, avian influenza mutates rapidly. Complete depopulation removes valuable food and economic resources from animals who must consume or sell their own birds for survival. These suggestions appear to be strictly empirical and not based on any modeling studies. In this work we develop modeling tools to investigate these alternative control methods.

Infectiousness, transmissibility, and death rate depends on viral load [11–15]. Recent experiments with chickens have begun to quantify this dependence for avian influenza [16]. We develop and analyze a viral titer structured transmission model that extends previous work (e.g. [17, 18], which were motivated by HIV transmission) in several important ways: (1) Our model allows for both direct transmission from infectious animals and indirect transmission from an environmental reservoir, which according to veterinarians, is frequently an essential mode of transmission. (2) We allow recovery from the disease. (3) We include a latent infection class, and (4) We incorporate a large variety of control measures, including antivirals, vaccination, isolation, environmental disinfection, and several forms of culling, which may result in fewer dead animals. We provide explicit formulae for the basic reproduction number, *R*_0_, for each intervention and for combinations.

We simulate a realistic outbreak of low pathogenic avian influenza (LPAI) on a mid-sized commercial turkey farm and evaluate the efficacy of the control methods.

## Basic model description

Many previous investigators have employed structured compartment transmission models, e.g., age-structured models, spatially structured models, contact risk structured, immunization status structured, but this appears to be one of the first viral titer structured transmission models. We recommend [11] for a general introduction to compartment transmission models.

**Fig 1:**
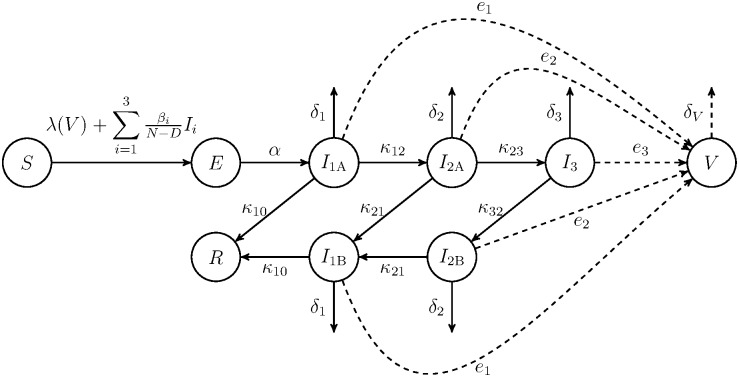
Basic viral titer compartment transmission model schematic with three infection classes. The rates of transfer between the classes are indicated, where *I*_1_ = *I*_1A_ + *I*_1B_ and *I*_2_ = *I*_2A_ + *I*_2B_.

We first construct a viral titer structured compartment transmission model, without interventions (see Fig 1 for a model schematic with three infected classes). We assume frequency dependent transmission by direct contact and indirect transmission via contact with an environmental reservoir. We make the simplifying assumption that there is a single susceptible species and a single strain of virus. We divide the population into susceptible *S*, exposed *E*, infectious *I*, and removed *R* animals. Including an exposed class allows an explicit consideration of the disease latency period. The removed class includes animals that are not susceptible and not infected. In latter sections, the removed class also contains vaccinated and isolated animals. Each infected animal sheds virus into the environment at a rate depending on its viral load. Since a virus requires a host to multiply, we assume no growth of viruses in the environment and, furthermore, that viruses in the environment lose their infectiousness over time.

We additionally split the infected animals into distinct subgroups based on their viral load; the differences in viral load result in each subgroup having their own transmission and death rates. Each infected animal progresses through the infected classes as their viral load increases. As their viral load decreases, the animal progresses down the chain of infected classes to the removed class. For each animal the viral load increases to some maximum level, which may be different per animal, though there is an absolute maximum level that no animal may surpass.

More precisely, we decompose the range of all possible viral titer levels into *n* subintervals. The animals with viral load in the *j*^*th*^ subinterval are in infected class *I*_*j*_. In the model we distinguish between those animals who are entering the *I*_*j*_ class for the first time, *I*_*j*A_, and those who are recovering, *I*_*j*B_. Referencing the notation in the model schematic, Fig 1, some animals will enter *I*_1*A*_ before recovering, while others will progress from *I*_1*A*_ to *I*_2*A*_ to *I*_1*B*_ before recovering, and so on. For the infected classes, we let *κ*_*ab*_ be the transfer rate from class *I*_*a*_ to neighboring class *I*_*b*_, which corresponds to an increase or decrease in the viral titer load.

Susceptible animals become infected through contact with animals in any infected class and through contact with virus in the environment. Susceptible animals who are infected move into the exposed class and become infectious at a rate *α*, moving into infectious class *I*_1*A*_. We assume that the transmission rate between animals depends on the viral load of the infecting animal; *β*_*j*_ is the rate of new infections generated by animals in class *I*_*j*_. We assume that the transmission rate is higher with a higher viral load, so that *β*_*i*_ < *β*_*i*+1_ for 1 ≤ *i* < *n*. The rate that animals become infected through contacts with the environment is determined by function *λ*(*V*). Two frequently used functions for *λ*(*V*) are the linear function *λV* and the Hill function *λV*/(*K* + *V*), where *K* is a positive constant.

Animals in infectious class *I*_*j*_ shed virus into the environment *V*, at rate *e*_*j*_. Virus in the environment lose infectiousness at rate *δ*_*V*_.

Infected animals in the first infected class, with the lowest viral load, recover at a prescribed rate *κ*_10_. We assume that the viral load is zero for animals in the susceptible and removed classes. Animals in the exposed class cannot infect others and do not shed virus into the environment. Recovery provides permanent immunity. Mortality for infected animals is included, with rates *δ*_*j*_ depending on the level of infection. Because we are modeling short-term outbreaks, we assume no natural births or deaths.

We do not include any immigration or emigration of animals into or from the population during the outbreak. Thus for domestic livestock populations there is no import or export of animals during the outbreak. The model assumes that the population mixes homogeneously. This is a reasonable assumptions for animals allowed to freely roam for some time each day such as free roaming broilers (chickens raised for their meat).

The viral titer structured compartment transmission model is given by the following system of ordinary differential equations.

$$\begin{array}{cc} \frac{dS}{dt} & =-\lambda \left(V\right)S-\sum_{i=1}^{n}\frac{\beta_{i}}{N-D}I_{i}S\\ \frac{dE}{dt} & =\lambda \left(V\right)S+\sum_{i=1}^{n}\frac{\beta_{i}}{N-D}I_{i}S-\alpha E\\ \frac{dI_{1A}}{dt} & =\alpha E-\kappa_{12}I_{1A}-\kappa_{10}I_{1A}-\delta_{1}I_{1A}\\ \frac{dI_{jA}}{dt} & =\kappa_{(j-1)j}I_{(j-1)A}-\kappa_{j(j+1)}I_{jA}-\kappa_{j(j-1)}I_{jA}-\delta_{j}I_{jA}\\ \frac{dI_{n}}{dt} & =\kappa_{(n-1)n}I_{(n-1)A}-\kappa_{n(n-1)}I_{n}-\delta_{n}I_{n}\\ \frac{dI_{jB}}{dt} & =\kappa_{(j+1)j}\left(I_{(j+1)A}+I_{(j+1)B}\right)-\kappa_{j(j-1)}I_{jB}-\delta_{j}I_{jB}\\ \frac{dI_{1B}}{dt} & =\kappa_{21}\left(I_{2A}+I_{2B}\right)-\kappa_{10}I_{1B}-\delta_{1}I_{1B}\\ \frac{dR}{dt} & =\kappa_{10}\left(I_{1A}+I_{1B}\right)\\ \frac{dD}{dt} & =\sum_{i=1}^{n}\delta_{i}I_{i}\\ \frac{dV}{dt} & =\sum_{i=1}^{n}e_{i}I_{i}-\delta_{V}V \end{array}$$

with initial conditions

$$S\left(0\right)=N-I_{0},\;I_{1A}\left(0\right)=I_{0},\;I_{1B}\left(0\right)=0,$$

$$E\left(0\right)=0,\;I_{j}\left(0\right)=0,\;R\left(0\right)=0,\;D\left(0\right)=0,\;V\left(0\right)=0,$$

for 1 < *j* < *n*, for total population of size *N*. All model parameters are positive (Table 1).

**Table 1:** Basic viral titer compartment transmission model parameters.

*β*_*i*_	transmission rate for *I*_*i*_
*α*	latency to infectious rate
*κ*_*ab*_	viral titer class change rate from *I*_*a*_ to *I*_*b*_
*δ*_*i*_, *δ*_*V*_	death rate for *I*_*i*_, *V*
*κ*_10_	recovery rate
*e*_*i*_	virus shedding rate for *I*_*i*_
*λ*(*V*)	environmental transmission rate function

Summing the left-hand sides of the differential equations for the number of animals gives the rate of change of the entire population and summing the right-hand sides gives 0. Therefore, the population size *N* is fixed, meaning,

$$N=S\left(t\right)+E\left(t\right)+\sum_{k=1}^{n}I_{k}\left(t\right)+R\left(t\right)+D\left(t\right),$$

for all *t* ≥ 0. Note that we include animals who die from infection *D* in the total population size.

To be most general, for the environmental infection rate *λ*(*V*) we require

- *λ*(0) = 0,
- *λ*(*V*) ≥ 0 when *V* > 0,
- *λ*(*V*) is differentiable at *V* = 0, and
- *λ*(*V*) ≤ *λ*′(0)*V*.

The first two conditions ensure that infection is possible from the environment only when there is virus present. The derivative of the infection rate appears in the expression for the basic reproduction number. The final technical condition is required to prove the global stability of the equilibrium point. (Refer to The Analysis of the Basic Model for details on the later two conditions.) The linear function *λ*(*V*) = *λV*, the Hill function *λ*(*V*) = *λV*/(*K* + *V*), and the function *λ*(*V*) = *ρ*(1 − *e*^−*φV*^) satisfy all conditions.

### The analysis of the basic model

It is easily seen that the only equilibrium points are the disease-free equilibrium points and they form a one-parameter family of equilibrium points

$$\left(S,E,I_{1A},\ldots ,I_{n},\ldots ,I_{1B},R,D,V\right)=\left(S^{*},0,\ldots ,0,R^{*},D^{*},0\right),$$

with 0 ≤ S* + R* + D* ≤ *N*. Given a particular initial condition, the equilibrium point corresponding to a particular initial condition depends on the choice of model parameters.

The model structure implies that each of *S*, *E*, *I*_*i*_, *R*, and *D* will be between 0 and *N*, and *V* will be between 0 and some maximum value depending on the size of the infected classes *I*_*i*_, and the parameters *e*_*i*_ and *δ*_*V*_.

#### The basic reproduction number, *R*_0_

The basic reproduction number is the number of secondary infections caused by each primary infection at the onset of the outbreak. When the basic reproduction number is less than one, then the outbreak will be driven to extinction. On the other hand, if the number is larger than one, the disease will begin to spread at an exponential rate. Our disease outbreak control strategies entail making interventions such that the resulting basic reproduction number is less than one.

Using the next generation operator method [19, 20], we compute the basic reproduction number for the general model with *n* infected classes.

**Theorem 1**. *For any number* *n* *of infected classes*, *the reproduction number is*

$$R_{0}=S_{0}\sum_{i=1}^{n}\left(B_{i}+e_{i}L\right)K_{01}\ldots K_{(i-1)i}\left(1+\sum_{j=1}^{n-i}\prod_{l=i}^{j+i-1}K_{l(l+1)}K_{(l+1)l}\right),$$ (1)

*where when* *i* = *n* *the final sum (from* *j* = 1 *to* *j* = 0*) is set to 0 and*

$$\begin{array}{c} B_{i}=\frac{\beta_{i}}{S_{0}+R_{0}},\;L=\frac{\lambda^{\prime }\left(0\right)}{\delta_{V}},\;K_{01}=\frac{1}{\delta_{1}+\kappa_{10}+\kappa_{12}},\\ K_{(i-1)i}=\frac{\kappa_{(i-1)i}}{\delta_{i}+\kappa_{i(i-1)}+\kappa_{i(i+1)}},\;K_{(n-1)n}=\frac{\kappa_{(n-1)n}}{\delta_{n}+\kappa_{n(n-1)}},\\ and\;K_{(i+1)i}=\frac{\kappa_{(i+1)i}}{\delta_{i}+\kappa_{i(i-1)}}, \end{array}$$

*for* 1 ≤ *i* < *n*.

As an example, for the model with three infected classes (Fig 1), the basic reproduction number is

$$\begin{array}{lll} R_{0}=S_{0} & ( & \left(B_{1}+e_{1}L\right)K_{01}\left(1+K_{12}K_{21}+K_{12}K_{23}K_{32}K_{21}\right)\\ & + & \left(B_{2}+e_{2}L\right)K_{01}K_{12}\left(1+K_{23}K_{32}\right)\\ & + & \left(B_{3}+e_{3}L\right)K_{01}K_{12}K_{23}). \end{array}$$

*Proof* *(Theorem 1)*. The infection system can be decomposed into new infections and all other movement out of and between infection classes. In particular for *n* = 3,

$${\left[\begin{array}{c} E\\ I_{1A}\\ I_{2A}\\ I_{3}\\ I_{2B}\\ I_{1B}\\ V \end{array}\right]}^{\prime }=\left[\begin{array}{c} \lambda \left(V\right)S+\sum_{i=1}^{n}\frac{\beta_{i}}{N-D}I_{i}S\\ 0\\ 0\\ 0\\ 0\\ 0\\ 0 \end{array}\right]-\left[\begin{array}{c} \alpha E\\ -\alpha E+\left(\kappa_{12}+\kappa_{10}+\delta_{1}\right)I_{1A}\\ -\kappa_{12}I_{1A}+\left(\kappa_{21}+\kappa_{23}+\delta_{2}\right)I_{2A}\\ -\kappa_{23}I_{2A}+\left(\kappa_{32}+\delta_{3}\right)I_{3}\\ -\kappa_{32}I_{3}+\left(\kappa_{21}+\delta_{2}\right)I_{2B}\\ -\kappa_{21}\left(I_{2A}+I_{2B}\right)+\left(\kappa_{10}+\delta_{1}\right)I_{1B}\\ -e_{1}I_{1}-e_{2}I_{2}-e_{3}I_{3}+\delta_{V}V \end{array}\right],$$ (2)

Compute the Jacobian matrix for each of the vectors of the right-hand side. We have

$$F=\left[\begin{array}{ccccccc} 0 & \frac{\beta_{1}}{S+R}S & \frac{\beta_{2}}{S+R}S & \frac{\beta_{3}}{S+R}S & \frac{\beta_{2}}{S+R}S & \frac{\beta_{1}}{S+R}S & \lambda^{\prime }(V)S\\ 0 & 0 & \ldots & & & \ldots & 0\\ \vdots & & & & & & \vdots \\ 0 & 0 & \ldots & & & \ldots & 0 \end{array}\right]$$

and

$$V=\left[\begin{array}{ccccccc} \alpha & 0 & 0 & 0 & 0 & 0 & 0\\ -\alpha & \kappa_{12}+\kappa_{10}+\delta_{1} & 0 & 0 & 0 & 0 & 0\\ 0 & -\kappa_{12} & \kappa_{21}+\kappa_{23}+\delta_{2} & 0 & 0 & 0 & 0\\ 0 & 0 & -\kappa_{23} & \kappa_{32}+\delta_{3} & 0 & 0 & 0\\ 0 & 0 & 0 & -\kappa_{32} & \kappa_{21}+\delta_{2} & 0 & 0\\ 0 & 0 & -\kappa_{21} & 0 & -\kappa_{21} & \kappa_{10}+\delta_{1} & 0\\ 0 & -e_{1} & -e_{2} & -e_{3} & -e_{2} & -e_{1} & \delta_{V} \end{array}\right].$$

The theory of the next generation matrix method allows one to compute the basic reproduction number as the largest positive eigenvalue of the product $FV^{-1}$. The product has the form

$$FV^{-1}=\left[\begin{array}{cccccc} R & ⋆ & ⋆ & ⋆ & ⋆ & ⋆\\ 0 & \ldots & & & \ldots & 0\\ \vdots & & & & & \vdots \\ 0 & \ldots & & & \ldots & 0 \end{array}\right],$$

each ⋆ is some positive expression of the model parameters. The six eigenvalues of this matrix are R, 0, 0, 0, 0, and 0, and the basic reproduction number is R given by Eq (1).

#### Global stability

We construct a global Lyapunov function implying that the disease free equilibrium point

$$\left(S,E,I_{1},\ldots ,I_{n},R,D,V\right)=\left(S^{*},0,\ldots ,0,R^{*},D^{*},0\right),$$

is globally asymptotically attracting when *R*_0_ < 1. (See [17] for similar argument.)

**Theorem 2**. *If* *R*_0_ < 1, *any equilibrium point with* S* > 0 *is globally asymptotically stable*. *Thus the total number of exposed and infected individuals will diminish to 0*, *as will the number of virus particles in the environment*.

*Proof*. For notational convenience, we reindex the infected classes as

$$I_{1A}=I_{1},\ldots ,I_{n}=I_{n},I_{(n-1)B}=I_{n+1},\ldots ,I_{1B}=I_{2n-1}.$$

The infection system (2) can be written in the form (with *n* = 3)

$${\left[\begin{array}{c} E\\ I_{1A}\\ I_{2A}\\ I_{3}\\ I_{2B}\\ I_{1B}\\ V \end{array}\right]}^{\prime }={\left[\begin{array}{c} E\\ I_{1}\\ I_{2}\\ I_{3}\\ I_{4}\\ I_{5}\\ V \end{array}\right]}^{\prime }=\left[\begin{array}{c} \lambda \left(V\right)S+\sum_{i=1}^{2n-1}\frac{\beta_{i}}{N-D}I_{i}S\\ 0\\ 0\\ 0\\ 0\\ 0\\ 0 \end{array}\right]-V\left[\begin{array}{c} E\\ I_{1A}\\ I_{2A}\\ I_{3}\\ I_{2B}\\ I_{1B}\\ V \end{array}\right],$$ (3)

Define vector

$$\begin{array}{cc} c & =(c_{0},c_{1},\ldots ,c_{n},\ldots ,c_{2n-1},c_{2n})\\ & =\left(0,B_{1},\ldots ,B_{n},\ldots ,B_{1},\lambda^{\prime }\left(0\right)\right)V^{-1} \end{array}$$

and note that $c_{0}=\frac{R_{0}}{S_{0}}$.

Consider the function $L=c_{0}E+\sum_{i=1}^{2n-1}c_{k}I_{k}+c_{2n}V$, the dot product of the vector **c** and the vector of infections [*E*, *I*_1_, …, *I*_2*n*−1_, *V*]. Taking the derivative of *L* and evaluating along a solution *S*(*t*) yields

$$\begin{array}{cc} L^{\prime } & =c_{0}E^{\prime }+\sum_{i=1}^{2n-1}c_{i}I_{i}^{\prime }+c_{2n}V^{\prime }\\ & =c_{0}\left(\lambda \left(V\right)S+\sum_{i=1}^{2n-1}\beta_{i}I_{i}S\right)-\sum_{i=1}^{2n-1}B_{i}I_{i}-\lambda^{\prime }\left(0\right)V\\ & \leq \left(c_{0}S_{0}-1\right)\left(\sum_{i=1}^{2n-1}B_{i}I_{i}+\lambda^{\prime }\left(0\right)V\right)\\ & =\left(R_{0}-1\right)\left(\sum_{i=1}^{2n-1}B_{i}I_{i}+\lambda^{\prime }\left(0\right)V\right). \end{array}$$

The inequality holds because of the fourth technical condition placed on *λ*(*V*) and since along solution trajectories the number of susceptible animals decreases from *S*(0) to S*.

## Mitigation strategies and recommendations

We consider various control strategies and incorporate each into the basic transmission model and calculate the resulting *R*_0_. We provide recommendations to force the basic reproduction number less than one.

### Vaccination

Vaccination can be used to prevent infection before an outbreak or to reduce the susceptible pool during an outbreak. Many farm animals, even in developing countries, are routinely vaccinated against endemic diseases such as Newcastle disease in poultry and Q fever in goats [21]. Emergency vaccinations are used during some outbreaks, however, some countries may not import animals given an emergency vaccination [22]. Also, emergency vaccinations require quick access to large quantities of strain-specific vaccines, which may be cost or time prohibitive.

Ideally, vaccination prevents a susceptible animals from ever being infected. However, few vaccines prevent infection entirely. Highly effective livestock vaccines typically reduce the likelihood and severity of clinical disease expression and reduce the amount of viral shedding. For the purposes of modeling, we assume vaccinated animals are moved from the susceptible to the removed class. Assuming that fraction *f* of the population is vaccinated at random and the vaccine has efficacy *e*, then fraction *f*_*v*_ = *ef* of the population is protected by the vaccination and the basic reproduction number is

$$\left(1-f_{v}\right)R_{0},$$

where *R*_0_ is the basic reproduction number for the base model (Eq (1)). Thus, to end the outbreak, a fraction *f* of the population is given the vaccine, where *f* is larger than

$$\left(1-\frac{1}{R_{0}}\right)\frac{1}{e}.$$

For a perfectly effective vaccine (*e* = 1), this is the well-known herd immunity threshold [11].

### Isolation

Isolation separates known infected animals from those not yet known to be infected. The rapid removal of sick and dead animals has long been a mainstay strategy to control outbreaks in livestock populations. For example, to control Pullorum disease, which was essentially eradicated many years ago in commercial flocks, a rapid blood test was used and reactors were immediately removed from the flock [23]. A similar approach has been used to control bovine brucellosis and tuberculosis, and equine infectious anemia [21]. However for large outbreaks, the logistics of isolation are frequently difficult to achieve in both human and animal populations.

Isolated animals are moved from their current infection class to the removed class. Animals can be moved all at once (rapid) or over time (gradual).

#### Targeted rapid isolation

Targeted rapid isolation involves testing the animals and isolating those that are currently in some number of the most infectious classes. One can affect this in two ways: isolating all animals in the highest infected classes or isolating only a fraction of those animals. We call the first complete and the second partial targeted rapid isolation. We note that the same fraction need not be removed from each class. Assuming that visibly sick animals are those with the highest viral loads and therefore in the most infectious classes, one may think of targeted rapid isolation as isolating all the animals assessed as having the most severe clinical signs.

Neither isolation strategy effects the value of the basic reproduction number since *R*_0_ does not depend on the number of animals in the infected classes and the population size *N* is fixed (see Eq (1)). While targeted isolation does not reduce *R*_0_, the dynamics of the outbreak will change. When the number of infected animals is smaller, the number of susceptible animals will decrease more slowly, perhaps allowing time for other interventions to be used. To see this multiply the size of the highest infected classes in the *dS*/*dt* equation by 1 − *f*_*c*_. Certainly we must have that

$$0>-\lambda \left(V\right)S-\sum_{k=1}^{j-1}\beta_{k}I_{k}S-\sum_{k=j}^{n}\beta_{k}I_{k}\left(1-f_{c}\right)S>-\lambda \left(V\right)S-\sum_{k=1}^{n}\beta_{k}I_{k}S.$$

A similar argument will hold if the fraction removed from each class is different.

#### Targeted gradual isolation

For targeted gradual (continuous) isolation, only animals in the highest infection classes are isolated. In the *R*_0_ formula replace *δ*_*i*_ by *δ*_*i*_ + *f*_*c*_ only for those classes that are isolated. For example, isolating from only the top class, *I*_*n*_, in the *R*_0_ formula, we have

$$K_{(n-1)n}=\frac{\kappa_{(n-1)n}}{\delta_{n}+f_{c}+\kappa_{n(n-1)}}.$$

### Environmental disinfection

Disinfection of surfaces to prevent transmission by contact is one of the cornerstones of disease transmission prevention. Many animal diseases are known to transmit via contact with virus particles present in the environment, such as FMD [24], equine infectious diseases [25], classic swine fever [21], and avian influenza [26]. For animals, environmental disinfection consists of disinfection of infected housing, equipment, vehicles, manure, or the removal of dead animals. Additionally, the guidelines recommended by the FAO & OIE and others to reduce the mass culling of infected poultry include careful disposal of infected animals and their products, as well as careful disinfection of the habitats of the infected animals (see for example [10]).

There are several ways to effect environmental disinfection, including one-time and continuous. These are similar to the isolation strategies of rapid and gradual, respectively.

#### One-time environmental disinfection

A one-time environmental disinfection rapidly reduces the pathogens present in the environment. The environment could be completely cleaned (*V* = 0) or only partially cleaned. In either case, environmental disinfection does not effect the value of the basic reproduction number *R*_0_, since *R*_0_ does not depend on the specific number of pathogens in the environment. However, the dynamics of the outbreak will change when the environment is disinfected, the number of susceptible animals will decrease more slowly. This follows from a similar argument as for targeted rapid isolation.

#### Continuous environmental disinfection

In the basic formula for *R*_0_, the term *δ*_*V*_ represents the rate at which the virus naturally degrades outside of a host, i.e. loses its infectivity. To account for the continual disinfection of the habitat, replace *δ*_*V*_ with a higher value representing the natural virus degradation plus the effect of the environmental disinfection, *δ*_*V*_ + *b*. As a result, in the *R*_0_ formula, the term in the formula for *R*_0_ coming from environmental infections becomes

$$L=\frac{\lambda^{\prime }\left(0\right)}{\delta_{V}+b}.$$

### Culling

Complete depopulation is the current method for control of many livestock viral diseases. However, there are several culling strategies that may result in fewer dead animals. These include culling only the animals assessed as having the most severe clinical signs, culling a fraction of randomly selected animals, or culling over a longer time scale (gradual). Some authors argue that culling over a longer time scale is likely to lead to increased rather than decreased animal health and welfare impacts [27]. Drawbacks to incomplete culling include continued disease presence, and economic issues, such as other countries being unwilling to import animals from places with recent outbreaks.

#### Untargeted rapid culling

One depopulation method is to randomly cull a fraction *f*_*c*_ of animals from the entire population at one time. Both susceptible and infected animals will be culled, a seemingly nonintuitive strategy. In this case, the resulting basic reproduction number is

$$\left(1-f_{c}\right)R_{0},$$

where *R*_0_ is the basic reproduction number for the basic model (Eq (1)), because the initial number of susceptible animals changes from *S*_0_ to *S*_0_(1 − *f*_*c*_). Thus, if a fraction *f*_*c*_ of the animals are culled larger than

$$1-\frac{1}{R_{0}},$$

then the outbreak will rapidly end. The above threshold is equal to the well-known herd immunity threshold for vaccination [11] and has a similar interpretation here.

#### Untargeted gradual culling

Gradual culling is the continuous culling of a small to moderate fraction of animals over a long time period, instead of a one-time rapid depopulation. Each day fraction *f*_*c*_ of the population is culled at random, which in the model equations is an additional disease related death that affects all classes, including *S* and *R*. Therefore, in the system of differential equations and the corresponding basic reproduction number each *δ*_*i*_ term is replaced with *δ*_*i*_ + *f*_*c*_. In the *R*_0_ formula, we have

$$\begin{array}{cc} K_{01} & =\frac{1}{\delta_{1}+f_{c}+\kappa_{10}+\kappa_{12}},\;K_{(i-1)i}=\frac{\kappa_{(i-1)i}}{\delta_{i}+f_{c}+\kappa_{i(i-1)}+\kappa_{i(i+1)}},\\ K_{(n-1)n} & =\frac{\kappa_{(n-1)n}}{\delta_{n}+f_{c}+\kappa_{n(n-1)}},\;\text{and}\;K_{(i+1)i}=\frac{\kappa_{(i+1)i}}{\delta_{i}+f_{c}+\kappa_{i(i-1)}}, \end{array}$$

for 1 ≤ *i* < *n*.

#### Targeted rapid culling

Targeted rapid culling involves testing the animals and culling those animals that are currently in some number of the most infectious classes. Unlike untargeted culling, no susceptible animals are culled. The effect of targeted rapid culling is exactly as the effect of targeted rapid isolation.

#### Targeted gradual culling

For targeted gradual (continuous) culling, only animals in the highest infection classes are culled. The effect of targeted gradual culling is exactly as the effect of targeted gradual isolation.

### Antivirals

Antiviral drugs disrupt the life cycle of the virus, reducing or halting its spread. Although there is a large arsenal of antivirals licensed for human use, at present, only one antiviral drug has been licensed for use in veterinary medicine, feline interferon-omega [28]. Regardless, several of the antivirals licensed for human use have been used for animal diseases. However, the extensive use of antiviral agents against the infection in swine and poultry could promote the emergence of resistant strains and impair the use of these drugs in humans. For this reason, the use of antivirals in animals is discouraged, e.g., since 2006 the FDA has prohibited the use of antivirals in poultry in the United States [29] (and see Code of Federal Regulations 21CFR530.41).

The use of antiviral drugs results in animals moving from an infected class with higher viral load to classes with lower load more quickly. Assuming all infected animals receive the antiviral at the same time, then the rate of transfer from a more infected class to a less infected class increases by rate *a*. Therefore, each *κ*_(*i*+1)*i*_ term in the system of differential equations and the basic reproduction number becomes *κ*_(*i*+1)*i*_ + *a*. If only *p*% of the animals in each class are given the antiviral, then *κ*_(*i*+1)*i*_ is replaced with *κ*_(*i*+1)*i*_ + *ap*. In the *R*_0_ formula (Eq (1)), we have

$$\begin{array}{cc} K_{(i-1)i} & =\frac{\kappa_{(i-1)i}}{\delta_{i}+\kappa_{i(i-1)}+ap+\kappa_{i(i+1)}},\\ K_{(n-1)n} & =\frac{\kappa_{(n-1)n}}{\delta_{n}+\kappa_{n(n-1)}+ap},\;\text{and}\;K_{(i+1)i}=\frac{\kappa_{(i+1)i}+ap}{\delta_{i}+\kappa_{i(i-1)}+ap}, \end{array}$$

for 1 ≤ *i* < *n*.

### Combined methods of control

In the past, combinations of methods have been employed to control outbreaks, such as vaccination and antivirals to control outbreaks of influenza in chickens [30]. The basic reproduction number for combinations of control methods are the combinations of modifications as described above. For example, using both vaccination and antivirals has a basic reproduction number of

$$\left(1-f_{v}\right)R_{0a},$$

where *R*_0*a*_ is the basic reproduction number when using antivirals. Similarly, using both untargeted rapid culling and vaccination has a basic reproduction number of

$$\left(1-f_{c}\right)\left(1-f_{v}\right)R_{0}.$$

Other combined basic reproduction numbers are computed similarly.

### Model simulation: Low Pathogenicity Avian Influenza (LPAI) outbreak in a turkey farm

A main novelty of the model developed here is the multiple infected classes defined by viral titer. We use the data presented of a highly controlled transmission study of LPAI in turkeys [31] to estimate model parameter values to simulate an outbreak of LPAI in a turkey farm containing 10,000 birds. We estimate the remaining parameters using values from the literature [32–34]. See Table 2. We use our model with two infectious classes (with no latent period) and an environmental reservoir to capture the heterogeneity of viral titre load and transmission routes. The environmental transmission function is *λ*(*V*) = *ρ*(1 − *e*^−*φV*^) [34]. The value of *ρ* was selected so that none (*ρ* = 0), one-quarter (*ρ* = 0.06), half (*ρ* = 0.2), three-quarters (*ρ* = 0.6), or all (*ρ* = 10,000) of the transmission occurs due to the environment (Table 3).

**Table 2:** LPAI simulation model parameters, values, and references.

Parameter	Value	Reference
*β*_1_	1.5	[31]
*β*_2_	3	[31]
*α*	0.5	[33]
*κ*_01_, *κ*_12_, *κ*_21_	0.33	[31]
*δ*_1_	0.034	[31]
*δ*_2_	0.04	[31]
*δ*_*V*_	0.5	[32]
*e*_1_, *e*_2_	10,000	[34]
*ρ*	0, 0.06, 0.2, 0.6, or 10,000	see description in text
*φ*	1	[34]

**Table 3:** Comparison of dynamics for varying levels of environmental transmission. The percentage of total infections caused by the environment is determined by the value of *ρ*. Total deaths are determined at *t* = 30 days. In all three cases, all turkeys become infected. *The number of infections caused by direct transmission is less than 0.001.

Percentage	*ρ*	Time of peak	Infections at peak	Total deaths
0	0	19	3,583	1,335
1/4	0.06	8.0	3,822	1,398
1/2	0.2	6.0	4,092	1,399
3/4	0.6	4.5	4,416	1,399
1*	10,000	2.9	4,716	1,400

We simulate various control measures, including all types of culling and environmental disinfection. No control measure appreciably decreases the total number of dead birds, and in many cases, culling substantially increases the number of dead birds (Tables 4 and 5). Even with thorough environmental disinfection, the environment quickly becomes recontaminated, preventing almost no infections.

**Table 4:** Comparison of dynamics for targeted rapid culling, varying the percent of culling. Only animals in the most infectious class *I*_2_ are culled. Culling occurs on day 4. One-half of infections are indirect (*ρ* = 0.2). Total deaths are determined at *t* = 30 days.

Percent	Day of peak	Infections at peak	Total deaths	Total culled	Total lost
1	6.4	3,362	1,230	882	2,112
3/4	6.3	3,542	1,272	662	1,934
1/2	6.2	3,723	1,314	441	1,755
1/4	6.1	3,906	1,357	220	1,577
0	6.0	4,092	1,399	0	1,399

**Table 5:** Comparison of dynamics for targeted rapid culling, varying the day of culling. All infectious animals are culled (animals in *I*_1*A*_, *I*_2_, *I*_1*B*_). One-half of infections are indirect (*ρ* = 0.2). Total deaths are determined at *t* = 30 days.

Day of cull	Day of peak	Infections at peak	Total deaths	Total culled	Total lost
2	6.7	3,525	1,219	1,220	2,439
3	7.2	2,917	1,059	2,281	3,340
4	4.0	3,264	910	3,264	4,174
5	5.0	3,895	814	3,895	4,709
6	6.0	4,092	787	4,092	4,879

## Discussion

We construct a virus titer structured compartment transmission model allowing for different transmission and death rates for animals with different viral loads. The model includes both direct and indirect transmission through contacts with infectious animals and the environmental reservoir, respectively. We then use this model to investigate control strategies to end disease outbreaks. These include vaccinations, isolation, environmental cleaning, forms of less indiscriminate culling, antivirals, and combinations. We explicitly compute the basic reproduction number for each of the eradication methods and some combinations. Mathematical theory guarantees that if the basic reproduction number is less than one then the outbreak will be driven to extinction.

Of course there are many simplifying assumptions behind this, and every, model, and maybe more for mathematically tractable models. Sometimes simple models are more useful than sophisticated models. We purposely designed our model to have a minimal number of parameters besides vital titers, for use especially in the early phases of an outbreak where little is known.

Although the nascent field of point-of-care instant molecular diagnostics is advancing at lightening speed (see for example the GeneXpert Omni, by Cepheid (www.cepheid.com/us/genexpert-omni, accessed 12/13/2016), at this time there appears to be a paucity of viral titer structured data from transmission studies or outbreaks available in the literature. We thank a referee for providing references [31] and [35]. With the improvement in rapid diagnostics, this model and control strategies could be used in nearly realtime.

Based on [31], we simulate an LPAI outbreak in a mid-sized turkey farm. No control strategy helped in any appreciable way and culling resulted in more total lost birds, significantly more when all infectious birds are culled. Beside prediction, a major use of modeling is to generate hypotheses. We hope the quantitative predictions in this manuscript will stimulate further research into the efficacy of culling to control outbreaks of infectious diseases in animal populations.

Our simulations show these control methods will work best for diseases that move relatively slowly through the animal populations, such as laryngotracheitis in poultry [21]. Avian influenza control in intensive (large and dense) commercial flocks presents formidable challenges. Highly pathogenic strains move rapidly within a flock, with many birds appearing sick or dying at the same time. For such outbreaks total depopulation (culling all birds) may remain the best strategy to control the outbreak. Economically, total depopulation may also be preferred, since poultry cannot be exported from an infected farm until the influenza is completely absent from the farm.

These models assume mass action mixing of the susceptible and infected animals. This is the usual null model in epidemiological models. The assumption of mass action mixing should be reasonable for any population of free-roaming animals, or at least free-roaming for part of the day, such as broiler, organic-pasture, or free-range chickens. The assumption may also be applicable for some viral infections spread via aerosol transmission, even if the animals themselves are confined in cages, such as laying hens. In general, the risk of airborne transmission varies dramatically in spatial and temporal proximity from infected animals [36].

A desirable consequence of the explicit formulas for the basic reproduction number is it is straightforward to compute the sensitivities and elasticities for each (see [37] as a reference). For example, in the combination of unbiased rapid culling, vaccination, and antivirals, the basic reproduction number has the same sensitivity with respect to culling and vaccination.

The transmission models we present apply to a single geographic location (patch). Modeling control strategies for FMD frequently involves multi-farm transport, possibly of contaminated equipment, which we do not consider. Spatially explicit modeling, e.g. including multiple locations and other spatial considerations, have been extensively studied in [8, 11, 38–42]. Additional extensions, such as the addition of disease carriers, natural births and deaths (for longer-term outbreaks), differential susceptibility by host species or viral strain, and explicit consideration of the economic cost of implementing the control strategies, can certainly be added to our basic model, and similar control strategies can be studied.
